# Supplementary material for: Characterization of Rhesus Macaque Embryonic Stem Cells in Primed and Naïve-like Cell States of Pluripotency Using Fourier Transform Infrared (FTIR) Microspectroscopy
Source: Int J Mol Sci. 2025 Sep 29;26(19):9514. doi: 10.3390/ijms26199514 (PMC12525272; doi:10.3390/ijms26199514)
Supplement: Supplementary file 1 [file ijms-26-09514-s001.zip › ijms-3850341-supplementary.pdf]

International Journal of
Molecular Sciences

MDPI

Supplementary Materials:

Table S1. Primary antibodies used for Immunocytochemistry.

Marker types	Primary antibodies	Origin	Dilution
**Core pluripotency markers**	*OCT4* (Santa Cruz Biotechnologies sc-9081)	Rabbit	1:100
	NANOG (R&D system AF1997)	Goat	1:100
	SOX2 (R&D system AF2018)	Goat	1:100
**Primed pluripotency marker**	*OTX2* (R&D system AF1979)	Goat	1:200
**Naïve pluripotency markers**	*TFAP2C* (R&D system AF5059)	Goat	1:100
	KLF17 (Atlas Antibodies HPA024629)	Rabbit	1:100
	ALPPL2 (abcam ab96947)	Rabbit	1:100
	TFCP2L1 (abcam ab123354)	Rabbit	1:100

Table S2. Secondary antibodies used for Immunocytochemistry

Secondary antibodies	Origin	Dilution
Alexa Fluor 647 (Invitrogen A21447) (Far red)	Donkey anti-Goat	1:500
Alexa Fluor 555 (Invitrogen A21432) (Red)	Donkey anti-Goat	1:500
Alexa Fluor 647 (Invitrogen A31573) (Far red)	Donkey anti-Rabbit	1:500
Alexa Fluor 555 (Invitrogen A31572) (Red)	Donkey anti-Rabbit	1:500

 https://doi.org/10.3390/ijms26199514

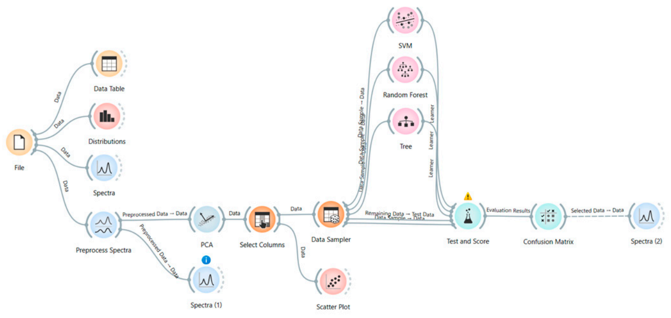

(**a**)

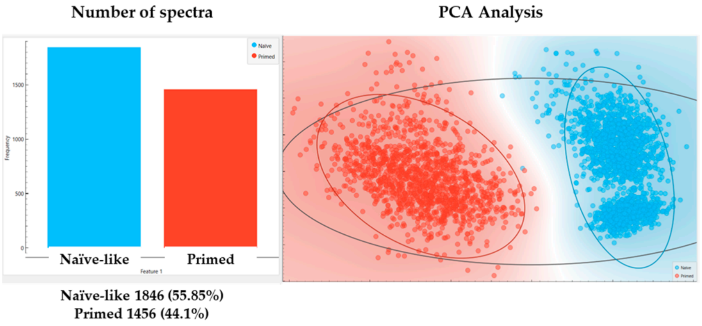

(**b**)

Test & Score Table

Evaluation results for target (None, show average over classes)

Model	AUC	CA	F1	Prec	Recall	MCC
Tree	0.999	0.998	0.998	0.998	0.998	0.997
Random Forest	1.000	0.998	0.998	0.998	0.998	0.997
SVM	1.000	0.998	0.998	0.998	0.998	0.997

(**c**)

SVM model

Tree
Random Forest
SVM

		Predicted		
		Naïve	Primed	Σ
Actual	Naïve	376	1	377
	Primed	0	284	284
	Σ	376	285	661

(**d**)

Figure S1. QUASAR: Machine Learning for cell classification. (**a**) A workflow for supervised analysis for rhESCs-FGF2/KOSR cells and rhESCs-ALGöX cells. (**b**) Number of spectra from rhESCs-FGF2/KOSR cells and rhESCs-ALGöX cells for PCA analysis. (**c**) The test and score table, shows cross-validated prediction quality of 3 methods: Tree, Random Forest and Support Vector Machine (SVM) (**d**) Confusion matrix from SVM model**.**
